# Revaluation of a selected segmental insufflation technique in total thoracoscopic lung segmentectomy

**DOI:** 10.1093/icvts/ivad054

**Published:** 2023-04-04

**Authors:** Jun Suzuki, Hiroyuki Oizumi, Hikaru Watanabe, Satoshi Takamori, Toshiya Fujiwara, Motoki Matsuura

**Affiliations:** Department of Surgery II, Faculty of Medicine, Yamagata University, Yamagata, Japan; Department of Thoracic Surgery, Higashiyamato Hospital, Tokyo, Japan; Department of Surgery II, Faculty of Medicine, Yamagata University, Yamagata, Japan; Department of Surgery II, Faculty of Medicine, Yamagata University, Yamagata, Japan; Department of Thoracic Surgery, Hiroshima City Organization Hiroshima City Hiroshima Citizens Hospital, Hiroshima, Japan; Department of Thoracic Surgery, Hiroshima City Organization Hiroshima City Hiroshima Citizens Hospital, Hiroshima, Japan

**Keywords:** Segmentectomy, Inflation–deflation lines, Inflation technique

## Abstract

We report selected insufflation technique using direct bronchial insufflation to visualize the intersegmental plane during total thoracoscopic segmentectomy. Following the transection of the bronchus using a stapler, a small incision was created in the dissected target bronchus, and direct air insufflation was performed at the small incision site. The target segment was inflated, while the preserved segments appeared to collapse, and a demarcating line was visualized between the inflated and deflated lung parenchyma. This technique quickly identifies the anatomic intersegmental plane without warranting special equipment such as jet ventilation or indocyanine green (ICG). Furthermore, this method saves time in creating inflation–deflation lines.

## INTRODUCTION

In recent years, anatomic segmentectomy is increasingly being considered to resection small, early-stage non-small-cell lung cancers. The important thing to performing anatomic thoracoscopic segmentectomy is an accurate delineation of the intersegmental line and comprehension of the segmental arteries and intersegmental veins, which are reliable landmarks of the intersegmental plane [1, 2]. The target segments’ inflation method benefits from obtaining a sufficient surgical margin from the tumour, and one of the most famous methods is high-frequency jet ventilation.

We previously reported creating an inflation–deflation line using a slip knot bronchial ligation method that could be useful for thoracoscopic segmentectomy [3]. This method is easy once one gets used to it, but it takes some time to delineate the inflation–deflation line. Previously, Fujiwara *et al.* reported a method of selectively inflating a segmental bronchus during hybrid VATS in Japan. Consequently, to solve the problems of the slip-knot method, we conducted the previously described method [4]. In this study, we investigate the effectiveness of the insufflation method in total thoracoscopic anatomic segmentectomy.

## PATIENTS AND METHODS

### Technique

The Ethics Committee of Yamagata University Hospital granted this study's ethics approval on 30 September 2022 (approval number: 2022-S-51); the requirement to obtain informed consent directly was waived. Surgery was performed with the patient in the lateral decubitus position under general anaesthesia and differential ventilation.

During the procedure, the surgeons stood on the ventral side of the patient, and the assistants stood on the dorsal side. The image on the side of the assistants was rotated 180 degrees. Four ports (1 with a diameter of 20 mm and 3 with a diameter of 5 mm) were prepared for the port-access technique by multiport, and a 3- to 4-cm operator's port was made between the middle axillary line and the anterior axillary line by uniport.

**Figure 1:**
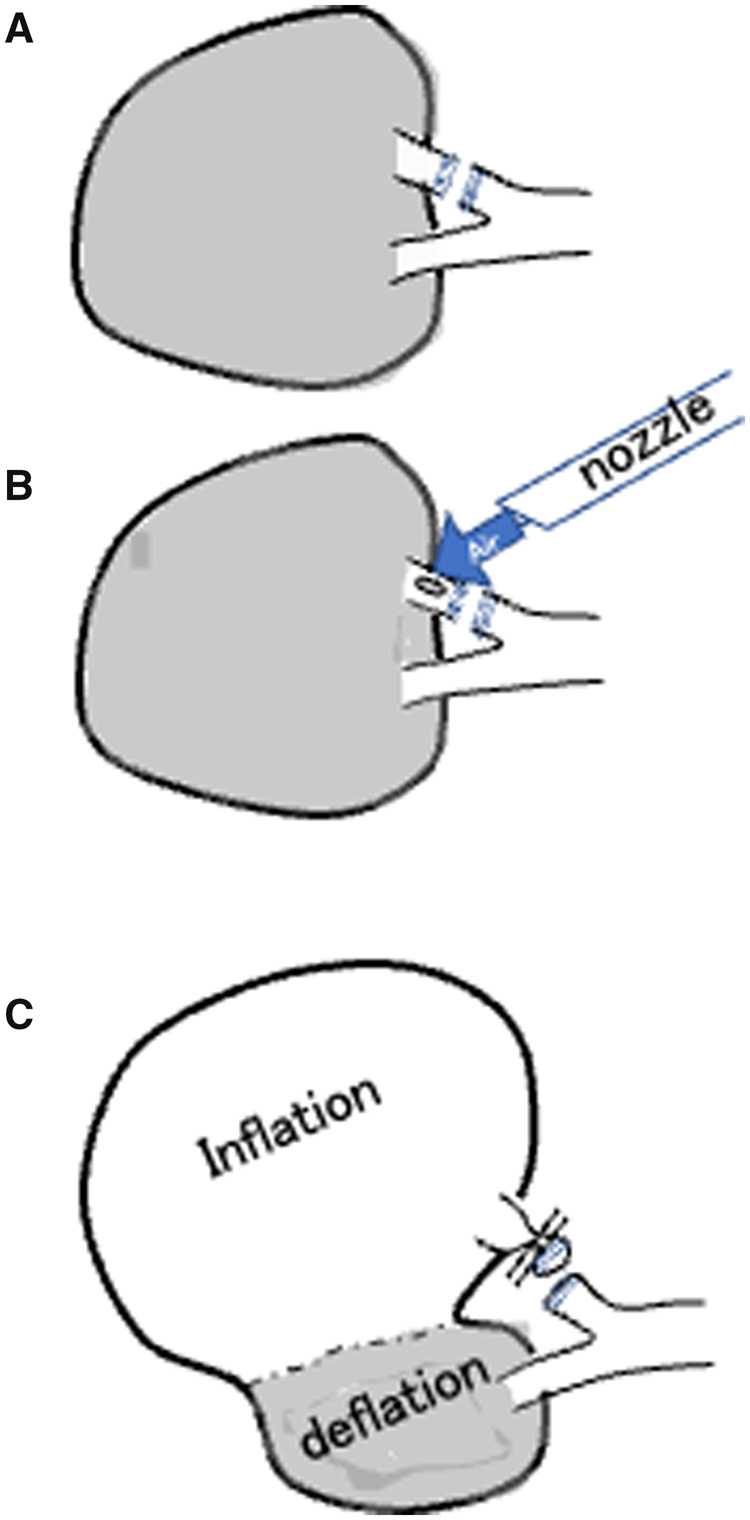
Distinguishing the intersegmental plane in thoracoscopic lung segmentectomy using a fibrin glue spray nozzle. (**A**) The bronchus is carefully divided with a stapler or ligated according to bronchial diameter. (**B**) After dissection of the bronchus, a small incision is made to the dissected target bronchus and direct air insufflation to the small incision site. After inflation of the air, the bronchus is closed using the clip. (This procedure is not always essential.) (**C**) The surgeon then dissects the parenchyma along the inflation–deflation line or the intersegmental vein using either electrocauterization or an energy device.

A rigid 5-mm 30 video thoracoscope was inserted. Thoracoscopic anatomic segmentectomy is initiated by dividing the lung parenchyma along an intersegmental vein. In addition, transection of the intra-segmental vein and artery under three-dimensional (3D) CT simulation is performed [1, 2]. After the transection of the target segment bronchus, we made a small incision to dissect the bronchus and performed direct air insufflation at the small incision using a commercially available fibrin sealant spray nozzle. The airflow pressure was ∼0.05 MPa and was adjusted according to the degree of lung inflation. While the preserved segments appeared to collapse, the target segment was promptly inflated, creating an inflation–deflation line between the target and preserved lung parenchyma (Fig. 1). The surgeon dissected the lung parenchyma along the inflation–deflation line or the intersegmental vein using electrocauterization or a tissue sealer (Video 1).

We performed this method in 37 cases between April 2019 and December 2022. The surgical approach was multiport in 20 patients, uniport in 16 patients, and robotic in 1. In all cases, the inflation–deflation lines were well delineated. In addition, there were no complications related to this procedure.

### Comment

Anatomic segmentectomy depends on the accurate delineation of the inflation–deflation line and comprehension of intersegmental veins, which are reliable landmarks of the intersegmental plane [1–4]. The intersegmental vein is located between segments, although considerable variations may be observed. Therefore, visualization of the inflation–deflation line is the gold standard for understanding the precise segmental plane. Selective jet ventilation into the affected segmental bronchus demarcates the inflation–deflation line and allows accurate surgical margin assessment [3]. Hence, we chose a method involving the expansion of the affected segment. However, this method requires another surgeon or anaesthetist to manoeuvre the bronchoscope and a specific device for jet ventilation.

Moreover, we found that unskilled anaesthetists could not insert the bronchoscope into the smaller bronchi, and certain institutions have experienced such difficulties. Therefore, various modifications have been devised. Direct inflation into the bronchus from the operative field using a butterfly needle was helpful. However, great care is warranted, as this approach may cause a fatal air embolism. We previously reported a slip-knot technique for bronchial ligation [1]. This present method is advantageous as it does not require special equipment and can be applied at any time. However, the slip-knot technique first requires bilateral ventilation, and the knot is tightened to block the outflow of segmental air. The pulmonary parenchyma will inflate, occupying substantial intrathoracic space; therefore, the operation is halted till the lung deflates.

Regarding the median time required for the slip-knot method, we reported that the technique requires 2.7 min for bilateral ventilation plus ∼10.6 min to visualize the intersegmental plane [5]. In contrast, the present method can direct insufflation, like the jet ventilation method, allowing us to promptly observe the inflation–deflation line with precision in a short time. We often use a commercially available fibrin sealant spray nozzle. However, the outer cannula of an indwelling vein needle may be used as an alternative. This method was first reported by Fujiwara *et al.* in 2009 [4]. However, this method was reported in Japanese journals and used in several institutions. When this paper was published, the hybrid VATS approach to segmentectomy was performed.

We reported our experience at several national and international conferences on the presentation of total thoracoscopic segmentectomy, where we received several inquiries about this method.

When this paper was published, thoracoscopic surgery was performed using a hybrid approach that combined small thoracotomy and direct visualization. However, this method could be applied to the current approaches, such as total thoracoscopic surgery.

The importance of segmentectomy has increased since then, and it is applicable not only in thoracoscopic surgery but also in robotic-assisted surgery.

A few important points should be considered when performing this procedure. The first concern is blowing at a short distance from the target bronchus. Excessive pressure may be applied if it wedges into the bronchus, causing barotrauma.

Second, if the airflow pressure is too high, the preserved lungs expand through Kohn's pore; therefore, care must be taken. Third, even though we intend to inflate the target segment, only the subsegment may be inflated because only the subsegment bronchus is blown.

In conclusion, distinguishing the intersegmental plane using the direct bronchial insufflation technique can rapidly create a precise intersegmental line.

This method is simple and can be applied in total thoracoscopic segmentectomy.

### Institutional review board approval and informed consent statement

The institutional review board approved this single-centre study of the Yamagata University Hospital (Project ID 2022-S-51). The patient provided written informed consent for the video presentation.
